# Supplementary material for: In Vitro Meiosis of Male Germline Stem Cells
Source: Stem Cell Reports. 2020 Nov 10;15(5):1140–53. doi: 10.1016/j.stemcr.2020.10.006 (PMC7664054; doi:10.1016/j.stemcr.2020.10.006)
Supplement: Document S1. Supplemental Experimental Procedures, Figure S1, and Table S1 [file mmc1.pdf]

Stem Cell Reports, Volume *15*

Supplemental Information

In Vitro Meiosis of Male Germline Stem Cells

Qijing Lei, Xin Lai, Jitske Eliveld, Susana M. Chuva de Sousa Lopes, Ans M.M. van Pelt, and Geert Hamer

Supplemental Information

In vitro meiosis of male germline stem cells

Qijing Lei[1], Xin Lai[1], Jitske Eliveld[1], Susana M. Chuva de Sousa Lopes[2], Ans M.M. van Pelt[1], Geert Hamer[1,*]

[1]Center for Reproductive Medicine, Reproductive Biology Laboratory, Amsterdam Reproduction and Development Research Institute, Amsterdam UMC, Location AMC, University of Amsterdam, 1105AZ, Amsterdam, the Netherlands

[2]Department of Anatomy and Embryology, Leiden University Medical Center, 2333 ZC, Leiden, Netherlands

*Corresponding address. Center for Reproductive Medicine, Reproductive Biology Laboratory, Amsterdam UMC, Location AMC, Meibergdreef 9, 1105 AZ, Amsterdam, The Netherlands. E-mail: g.hamer@amsterdamumc.nl

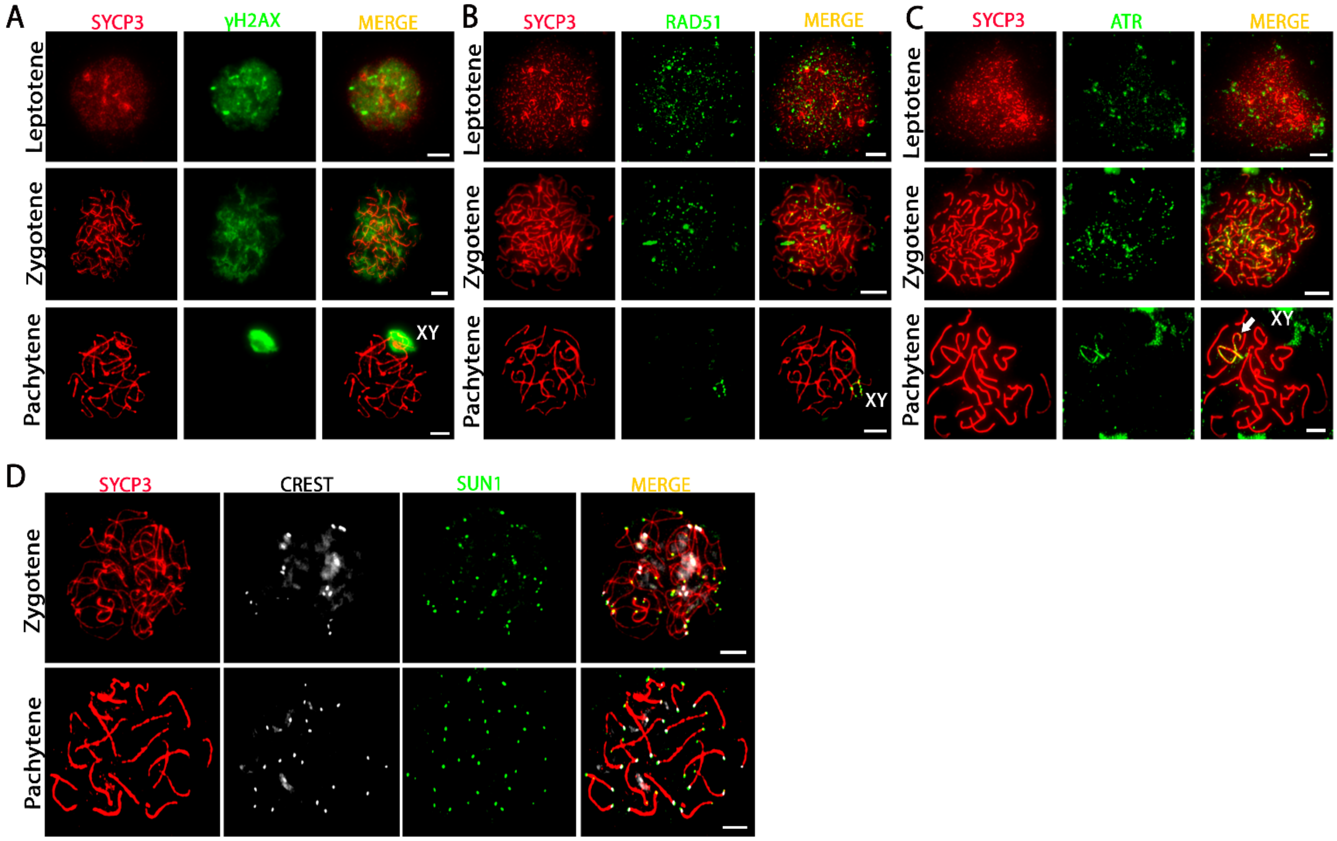

Figure S1. Positive control experiments using mouse testicular cells.

In vivo meiotic cells stained for SYCP3 (red) and **(A)** γH2AX (green), **(B)** RAD51 (green), **(C)** ATR (green), **(D)** SUN1 (green) and centromeres (CREST, white). Scale bar, 5µm.

Supplemental Experimental Procedures

**Primary antibodies**				
Target Protein	Host	Source	Cat. Number	IHC Dilution
γ-H2AX	Mouse	Merck Millipore	05–636	1:10,000
SYCP3	Mouse	Abcam	ab97672	1:600
SYCP3	Rabbit	NOVUS	NB300-231	1:2500
CREST-serum	Human	FitzGerald	90C-CS1058	1:600
ATR	Rabbit	Cell Signaling Technology	#2790	1:100
α-Tubulin	Mouse	Sigma	T9026	1:200
RAD51	Rabbit	Thermo Fisher Scientific	PA5-27195	1:100
MLH1	Mouse	BD Pharmingen	550838	1:50
SUN1	Guinea pig	Provided by Manfred Alsheimer	N/A	1:600
**Secondary antibodies**				
Fluorescence	Host	Source	Cat. Number	IHC Dilution
Alexa Fluor 488	Donkey anti-Mouse	Thermo Fisher Scientific	A21202	1:1000
Alexa Fluor 488	Donkey anti-Rabbit	Thermo Fisher Scientific	A21206	1:1000
Alexa Fluor 488	Goat anti-Guinea pig	Thermo Fisher Scientific	A11073	1:1000
Alexa Fluor 555	Goat anti-mouse	Thermo Fisher Scientific	A21424	1:1000
Alexa Fluor 555	Donkey anti-Rabbit	Thermo Fisher Scientific	A31572	1:1000
Alexa Fluor 647	Goat anti-Human	Thermo Fisher Scientific	A21445	1:1000

Table S1. Antibodies used in this study. Related to Figures 1, 3, 4, 5 and S1.

Supplemental Experimental Procedures

GS cells and Sertoli cell line culture

Mouse GS cells were cultured as previously reported (Kanatsu-Shinohara et al., 2003; Mulder et al., 2017; Zheng et al., 2017). After the third passage, the cells were cultured on mitotically inactivated mouse embryonic fibroblasts (MEFs; Gibco, A34962), using in a medium mainly composed of StemPro-34 SFM medium (Thermo Fisher Scientific), StemPro-34 Supplement (Thermo Fisher Scientific), 1% fetal bovine serum (FBS), recombinant human GDNF (10 ng/ml, 450-10, Peprotech), recombinant human bFGF (10 ng/ml, 100-18B, Peprotech), recombinant human EGF (20 ng/ml, AF-100-15, Peprotech), recombinant human LIF (10 ng/ml, CYT-644, Prospec), as well as other components as previously reported (Kanatsu-Shinohara et al., 2003). GS cells were refreshed every 2-3 days, dissociated by accutase (Thermo Fisher Scientific) and passaged every 5-7 days (doubling time, 3 days) at a ratio of 1:4-6 on fresh mitotically inactivated mouse embryonic fibroblasts. The cells were maintained at 37°C in 5% CO_2 in air. The cells used for this research were mostly at passage 20, with a maximum of 26 passages.

As a feeder cell, we used available Sertoli cell lines SK49 or TM4. SK49 was established by Walther et al., from 10-day-old male H-2Kb- tsA58 transgenic mice carrying an inducible temperature-sensitive SV40 T antigen (Walther et al., 1996). This cell line is able to express Sertoli cell-specific pattern markers and exhibit distinct Sertoli cell properties. TM4 was establishes by Matfier et al., from 11-13 days of age male BALB/c - nu/+ mice (Matfier, 1980). Both Sertoli cell lines were cultured at 37°C and 5% CO_2 in Dulbecco's Eagle's medium (DMEM; Thermo Fisher Scientific, Waltham, MA, USA) supplemented with 10% fetal bovine serum (FBS), penicillin (100 U/mL) and streptomycin (100 U/mL).

In vitro meiosis of GS cells

SK49 cells or TM4 Sertoli cells, inactivated by mitomycin (10µg/ml, M7949, Sigma), were grown on 12-well plates pre-coated with laminin (20 µg/ml, L2020, Sigma) to a density of 1 x10^5. Then GS cells were seeded on these Sertoli cells for two days to maintain GS cell proliferation as described previously (Kanatsu-Shinohara et al., 2003). To induce meiosis, day 0 to day 3 (Fig. 1A), the cells were cultured in a medium composed of StemPro-34 SFM medium and StemPro-34 Supplement, 10% KnockOut Serum Replacement (KSR), Retinoic acid (2µM, R2625, sigma), Recombinant Mouse BMP-4 Protein (20 ng/ml, 5020-BP, R&D Systems), Recombinant Mouse Activin A Protein (100 ng/ml, 338-AC, R&D Systems). From day 3 to day 9 after meiosis induction, the medium was composed of StemPro-34 SFM medium and StemPro-34 Supplement, 10% KSR, Bovine Pituitary Extract (BPE) (50µg/ml, 354123, Corning Life Sciences), Follicle-stimulating hormone (FSH) (200 ng/ml, F4021, Sigma), Testosterone (10 µM, 86500, Sigma) and refreshed daily.

Okadaic acid (OA)-induced generation of metaphase-like cells in vitro

For generation of metaphase-like cells *in vitro*, cells were cultured in Dulbecco's modified Eagle's medium (DMEM) supplemented with 5% FBS, 3 µM okadaic acid (OA; 459618, Millipore) at 34°C for 4 hours and fixed in 4% paraformaldehyde (PFA). For negative controls no OA was added.

Cytospins

The cells were detached from the culture dish using 0.25% trypsin and washed with 1x phosphate buffered saline (PBS) and diluted in 200 µL PBS/1% BSA containing 30,000 to 50,000 cells for each cytospin spot and spun for 7 minutes at 112g. The slides were air dried for 10min, fixed in 4% PFA and stored at 4°C in PBS or stored at -80°C after air drying.

Karyotyping

The Cells were dissociated into single cell suspension after 8 days after spermatogonial differentiation, subjected to hypotonic treatment with 75 mM KCl at 37 ◦C for 10 minutes and fixed in freshly prepared methanol/acetic acid (ratio: 3:1). Cells were dropped onto glass slides from 1 m distance and air dried. Chromosomes were stained with Hoechst 33342.

Flow Cytometry

For FACS analysis, after the cells were fixed and permeabilized in 70% Ethanol (EtOH). The cells were labeled with 1 µg/1 × 10^5 cells propidium iodide (PI, Sigma, P4964) in FACS/EDTA buffer (1× PBS with 1% FCS/0.1% NaN3/2 mM EDTA) containing DNAse-free RNAse A (v/v 1:20) for 5 min. The measurements and sorting were performed on a Sony SH800Z cell Sorter (Sony Biotechnology Inc. Japan). Data was analyzed using FlowJo software version 10. For acrosome detection, the sorted "haploid" peak (putative 1C-region) cells were incubated with lectin peanut agglutinin (PNA) conjugated with Alexa Fluor 488 conjugate (1:700, L21409, Life Technologies) for 15min.

Supplemental References:

Kanatsu-Shinohara, M., Ogonuki, N., Inoue, K., Miki, H., Ogura, A., Toyokuni, S., and Shinohara, T. (2003). Long-term proliferation in culture and germline transmission of mouse male germline stem cells. Biology of reproduction *69*, 612-616.

Matfier, J.P. (1980). Establishment and characterization of two distinct mouse testicular epithelial cell lines. Biology of reproduction *23*, 243-252.

Mulder, C.L., Catsburg, L.A.E., Zheng, Y., de Winter-Korver, C.M., van Daalen, S.K.M., van Wely, M., Pals, S., Repping, S., and van Pelt, A.M.M. (2017). Long-term health in recipients of transplanted in vitro propagated spermatogonial stem cells. Human reproduction *33*, 81-90.

Walther, N., Jansen, M., Ergün, S., Kascheike, B., and Ivell, R. (1996). Sertoli Cell Lines Established fromH-2Kb-tsA58 Transgenic Mice Differentially Regulate the Expression of Cell-Specific Genes. Experimental cell research *225*, 411-421.

Zheng, Y., Jongejan, A., Mulder, C.L., Mastenbroek, S., Repping, S., Wang, Y., Li, J., and Hamer, G. (2017). Trivial role for NSMCE2 during in vitro proliferation and differentiation of male germline stem cells. Reproduction *154*, 81-95.
