# Supplementary material for: In Vitro Meiosis of Male Germline Stem Cells
Source: Stem Cell Reports. 2020 Nov 10;15(5):1140–53. doi: 10.1016/j.stemcr.2020.10.006 (PMC7664054; doi:10.1016/j.stemcr.2020.10.006)
Supplement: Document S2. Article plus Supplemental Information [file mmc2.pdf]

Stem Cell Reports
Article

OPEN ACCESS

In Vitro Meiosis of Male Germline Stem Cells

Qijing Lei,[1] Xin Lai,[1] Jitske Eliveld,[1] Susana M. Chuva de Sousa Lopes,[2] Ans M.M. van Pelt,[1] and Geert Hamer[1,*]

[1]Center for Reproductive Medicine, Reproductive Biology Laboratory, Amsterdam Reproduction and Development Research Institute, Amsterdam UMC, Location AMC, University of Amsterdam, Meibergdreef 9, 1105 AZ, Amsterdam, the Netherlands
[2]Department of Anatomy and Embryology, Leiden University Medical Center, 2333 ZC, Leiden, the Netherlands
*Correspondence: g.hamer@amsterdamumc.nl
https://doi.org/10.1016/j.stemcr.2020.10.006

SUMMARY

In vitro spermatogenesis has been achieved by culturing mouse embryonic stem cells (ESCs) together with a cell suspension of male juvenile gonad. However, for human fertility treatment or preservation, patient-specific ESCs or juvenile gonad is not available. We therefore aim to achieve *in vitro* spermatogenesis using male germline stem cells (GSCs) without the use of juvenile gonad. GSCs, when cultured on immortalized Sertoli cells, were able to enter meiosis, reach the meiotic metaphase stages, and sporadically form spermatid-like cells. However, the *in vitro*-formed pachytene-like spermatocytes did not display full chromosome synapsis and did not form meiotic crossovers. Despite this, the meiotic checkpoints that usually eliminate such cells to prevent genomic instabilities from being transmitted to the offspring were not activated, allowing the cells to proceed to the meiotic metaphase stages. *In vitro*-generated spermatid-like cells should thus be thoroughly investigated before being considered for clinical use.

INTRODUCTION

An estimated 10%–15% of couples suffer from subfertility, of which roughly 50% are diagnosed with male factor infertility (Kumar and Singh, 2015). Almost 7% of all men are subfertile or infertile in their reproductive age, of which approximately 10%–15% are not able to generate functional spermatozoa (Hamada et al., 2013; Krausz, 2011). In many cases, elongated spermatids can be retrieved from a testis biopsy by testicular sperm extraction, followed by intracytoplasmic sperm injection. However, when the process of spermatogenesis itself is disturbed or absent, causing a total absence of haploid spermatids, no current treatment options are available.

Although far from human application, several attempts have therefore been made to recapitulate spermatogenesis *in vitro*. This was achieved by complete *in vitro* differentiation of embryonic stem cells (ESCs) (Easley IV et al., 2012; Geijsen et al., 2004; Nayernia et al., 2006; Zhou et al., 2016) or by first differentiating mouse ESCs to primordial germ cell-like cells (PGCLCs), spermatogonial stem cell-like cells, or germline stem cell (GSC)-like cells *in vitro*, which can undergo spermatogenesis after transplantation into the seminiferous tubules of infertile mice (Hayashi et al., 2011; Ishikura et al., 2016; Li et al., 2019). Although all these studies have reported the generation of haploid spermatid-like cells, only one study (Zhou et al., 2016) was able to recapitulate most key events that characterize successful meiosis and generation of fertile haploid germ cells *in vitro* (Handel et al., 2014). This was achieved by differentiation of mouse ESCs to PGCLCs, followed by co-culture with a suspension of neonatal testicular cells (Zhou et al., 2016). However, even in a future clinic, ESCs comprising the patient's own genetic material will most likely not be available for an adult human patient.

One possible alternative strategy is the *in vitro* generation of human PGCLCs (Kojima et al., 2017; Sasaki et al., 2015) or functional sperm (Easley IV et al., 2012; Eguizabal et al., 2011) from induced pluripotent stem cells (iPSCs) derived from one of the patient's own somatic tissues (Hendriks et al., 2015a, 2015b). However, although the contribution of iPSCs to the field of *in vitro* gametogenesis cannot be underestimated, the generation of iPSCs still requires a level of genetic reprogramming, of which the safety is currently not sufficiently investigated. Moreover, following current germ cell differentiation protocols in mice (Zhou et al., 2016), this would still require the use of a compatible human neonatal testis.

Importantly, in many cases, for instance, when the absence of haploid spermatids is caused by meiotic arrest (Jan et al., 2018), the patient still has spermatogonial stem cells (SSCs). SSCs are adult male GSCs that, via a perfect balance between self-renewal and differentiation, ensure lifelong sperm production. For these patients, an alternative option to restore fertility would be to use their own SSCs. Recently, a study reported that autologous grafting of cryopreserved prepubertal testis led to sperm production and offspring in a rhesus macaque (Fayomi et al., 2019). Also, *ex vivo* culture of testicular grafts of neonatal mouse testes (Sato et al., 2011a), cryopreserved neonatal mouse testis tissues (Yokonishi et al., 2014), or immature/mature mouse testes as hosts transplanted with SSCs (Sato et al., 2011b) resulted in the production of functional sperm. However, complete *in vitro* spermatogenesis in cultured adult human testicular fragments has not yet been achieved (Medrano et al., 2018; Portela

 © 2020 The Author(s).
This is an open access article under the CC BY license (http://creativecommons.org/licenses/by/4.0/).

et al., 2019a). Meanwhile, human prepubertal (Sadri-Ardekani et al., 2011) and adult (Sadri-Ardekani et al., 2009) SSCs can already be cryopreserved and propagated *in vitro*, which could enable their clinical application. One theoretical option would be to restore the mutation that causes spermatogenic arrest in SSCs followed by autotransplantation (Mulder et al., 2016). However, in addition to ethical issues concerning germline genome editing and the practical issue of the remaining uncorrected SSCs in the testis, the genetics behind spermatogenic failure are not known in most cases. Although still far away from clinical application, a way to circumvent this would be to differentiate SSCs *in vitro* to generate functional sperm (Sun et al., 2018). One study used mouse SSCs to generate a multipotent adult GSC line (maGSCs) that could be induced to differentiate into haploid male germ cells via the pluripotent ESC pathway (Nolte et al., 2010). Another study described the generation of mouse spermatids from a telomerase-immortalized spermatogonial cell line (Feng et al., 2002). However, because pluripotent cell lines are not clinically usable, direct induction of primary SSCs would be preferable. As one of the initial steps in preclinical research, we here describe a protocol for mouse *in vitro* meiosis that, to avoid the use of cell lines, iPSCs, or ESC-like cells, directly uses primary isolated mouse SSCs maintained in culture as male GSCs (Kanatsu-Shinohara et al., 2003). As described (Kanatsu-Shinohara et al., 2003), in our laboratory these GSCs also retain their stem cell capacity and are able to undergo full spermatogenesis and generate healthy offspring after transplantation into the testes of recipient mice (Mulder et al., 2017). Moreover, by using retinoic acid (RA) treatment, we are able to induce spermatogonial differentiation *in vitro*, which is an important early step of spermatogenesis that irrevocably pushes the spermatogonia away from self-renewal in the direction of meiosis (Zheng et al., 2018). *In vivo*, spermatogonial differentiation and meiosis are supported by growth factors and hormones that are secreted by the Sertoli cells (De Gendt et al., 2004; Ramaswamy and Weinbauer, 2014). Therefore, we used Sertoli cell lines with thoroughly investigated phenotypes and characteristics (Matfier, 1980; Walther et al., 1996) as a feeder layer to support the induction of meiosis *in vitro*. This not only circumvents the need for neonatal testicular tissue, which would interfere with possible future clinical application, but also avoids potential contamination with Leydig cells, or even remaining germ cells, during the isolation of Sertoli cells. Moreover, it also reduces the need for animal experimentation. Using fluorescence microscopy, karyotyping, and flow cytometry, we monitored meiotic progression and subsequent germ cell development. We conclude that, although several studies demonstrate mouse meiosis to occur *in vitro*, the function of DNA damage repair and meiotic checkpoints should be further investigated before human application can be considered.

RESULTS

In Vitro Meiosis on a Feeder Layer of Immortalized Sertoli Cells

In line with previous reports (Dann et al., 2008; Wang et al., 2016), we also recently characterized RA-induced spermatogonial differentiation (Zheng et al., 2018). Western blot, qPCR, and RNA-sequence analyses showed substantial downregulation of the SSC self-renewal genes *Plzf* and *Oct4*, while RNA and protein levels of the differentiation marker STRA8 markedly increased after 3 days of RA exposure. As published previously (Wang et al., 2016), we also observed that, when induced to differentiate using RA and grown *in vitro* on a feeder layer of mouse embryonic fibroblasts (MEFs), GSCs can develop into zygotene spermatocytes and, occasionally, even form pachytene-like spermatocytes. However, further germ cell development does not occur using this culture system.

With the aim to facilitate complete meiosis *in vitro*, we cultured the cells at 34°C and chose to use the Sertoli cell line SK49 as a feeder layer to support *in vitro* germ cell development. In addition, to better mimic the *in vivo* microenvironment, the culture plates were precoated with laminin. The culture system consisted of three stages, with three different culture media, that represent (1) spermatogonial self-renewal/proliferation, (2) SSC differentiation and early meiosis, and (3) meiosis and formation of spermatid-like cells (Figures 1A and 1B). During the first stage the GSCs were cultured with the factors known to be necessary for maintaining SSC self-renewal, glial-derived neurotrophic factor (GDNF), basic fibroblast growth factor (bFGF), and epidermal growth factor (EGF) (Kanatsu-Shinohara et al., 2003), and behaved exactly as described previously (Zheng et al., 2018). Also, spermatogonial differentiation was induced exactly as reported previously by us (Zheng et al., 2018) by replacing the medium with medium containing RA, activin A, and bone morphogenetic protein 4 (BMP4). Finally, to establish meiotic progression, the medium was replaced with medium containing follicle-stimulating hormone (FSH), testosterone, and bovine pituitary extract (BPE) to induce meiotic progression and subsequent spermatid formation.

To monitor germ cell development, we harvested cells 0, 1, 3, 5, 7, and 9 days after spermatogonial differentiation and visualized DNA, the synaptonemal complex, centromeres, and acrosome formation using DAPI; antibodies against SYCP3; CREST serum; and peanut agglutinin (PNA) (Figures 1C and 1D). Most stages of meiosis could be observed *in vitro*, including leptotene, zygotene,

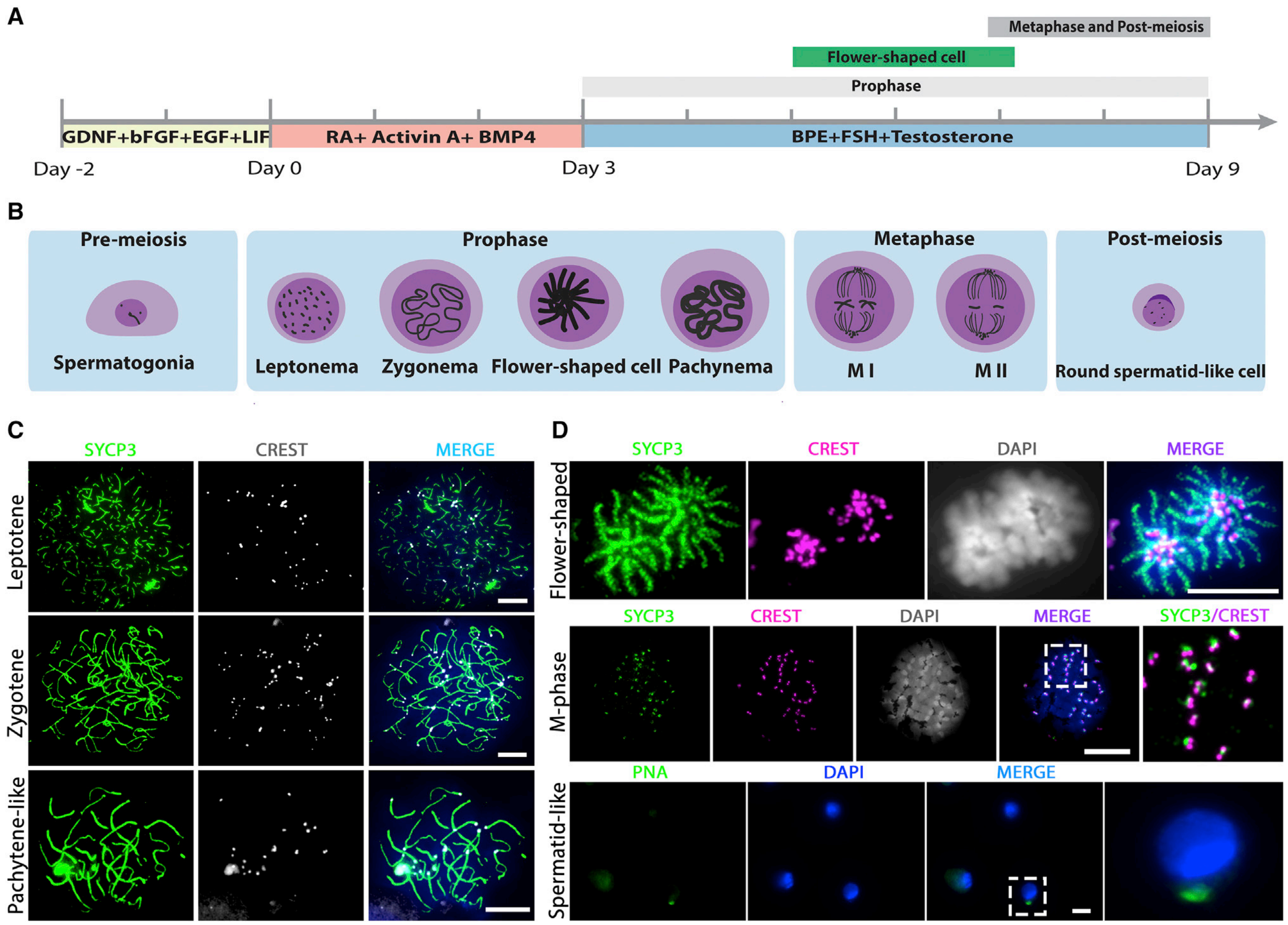

Figure 1. GSCs Undergo Meiosis *In Vitro* on a Feeder Layer of Immortalized Sertoli Cells

(A) Schematic overview of the three-step *in vitro* meiosis culture system. Bars above the timeline represent the period of relatively highly abundant presence of meiotic prophase I, flower-shaped, and meiotic M-phase/post-M-phase cells.

(B) Graphic of the different stages of *in vitro* germ cell development.

(C) Different stages of the meiotic prophase *in vitro*, including leptotene, zygotene, and pachytene-like, stained for SYCP3 (green), centromeres (CREST, white), and DNA (DAPI, blue).

(D) *In vitro*-generated flower-shaped, meiotic M-phase, and spermatid-like cells, stained for SYCP3 (green), centromeres (CREST, red), PNA (green), and DNA (DAPI, white/blue in merged). Regions inside dashed boxes are shown at higher magnification in the following image. Scale bars, 10 μm.

pachytene-like, meiotic metaphase (M-phase) cells, and, although rarely, spermatid-like cells (Figures 1B–1D). At day 3, leptotene cells appeared, and the first zygotene cells could be observed at day 3 (Figure 2A). At day 5, SYCP3-positive cells with "flower-shaped" chromosome morphology started to appear (Figures 1B, 1D, and 2A), which, to our knowledge, have not been reported before. Pachytene-like cells, although very rarely, also started to appear at day 5, while meiotic M-phase cells were present at day 7 and day 9 (Figure 2A). In contrast to mitotic M-phase cells, meiotic M-phase cells are characterized by SYCP3 staining at their centromeres, which promotes biorientation of the homologous chromosomes before chromosome segregation (Bisig et al., 2012). Spermatid-like cells could be observed occasionally after day 7. However, this cell type was observed only rarely, at the rate of one to three cells per microscope slide, and was mostly absent.

In Vitro Meiosis Depends on the Type of Sertoli Cells and Temperature

To investigate the influence of different Sertoli cell lines and different temperatures on *in vitro* spermatogenesis, we repeated the *in vitro* experiments using a feeder layer of either SK49 or TM4 Sertoli cells at 34°C or 37°C. Hence, four culture conditions, namely 34°C-SK49, 34°C-TM4, 37°C-SK49, and 37°C-TM4, were compared. Cells were

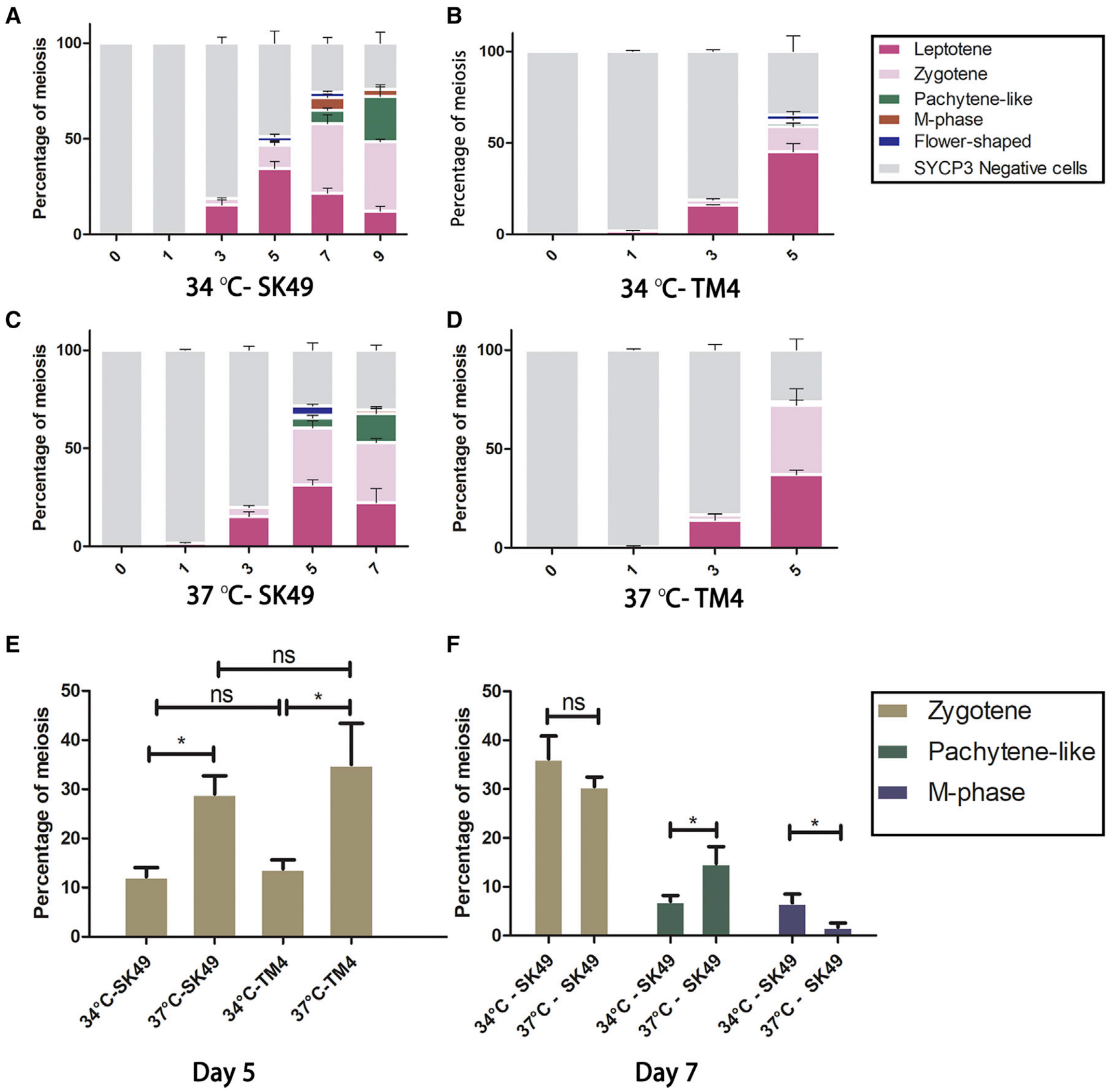

Figure 2. GSCs Undergo Meiosis *In Vitro* Most Efficiently at 34°C on SK49 Sertoli Cells

(A–D) Quantification of different cell types of *in vitro* gametogenesis at (A) 34°C on SK49 Sertoli cells, (B) 34°C on TM4 Sertoli cells, (C) 37°C on SK49 Sertoli cells, and (D) 37°C on TM4 Sertoli cells.

(E) Percentage of zygotene cells at day 5 at 34°C on SK49 Sertoli cells, 34°C on TM4 Sertoli cells, 37°C on SK49 Sertoli cells, and 37°C on TM4 Sertoli cells.

(F) Percentage of zygotene, pachytene-like, and meiotic M-phase cells at day 7 at 34°C and 37°C on SK49 Sertoli cells. Data are presented as the mean ± SEM, n = 3. *$p < 0.05$.

again harvested at 0, 1, 3, 5, 7, and 9 days of culture and studied using immunocytochemistry. The proportion of each cell type was quantified (Figures 2A–2D). Because many germ cells died after 4 days when grown on TM4 Sertoli cells, and the number of meiotic cells was too rare to count after 5 days, we stopped after day 5 in the TM4 group. For the same reason, the experiment was stopped after 7 days in the 37°C-SK49 group. We found that GSCs could be cultured for a longer time and had a higher differentiation efficiency in the SK49 group at both 34°C and 37°C (Figures 2A and 2C). Only about 2% of pachytene-like cells could be detected and less than 1% M-phase cells could be observed by co-culturing with TM4 at 34°C (Figure 2B), while no meiotic progression beyond zygotene could be observed in the 37°C-TM4 group (Figure 2D).

To assess the efficiency of early meiotic progression in all groups, we compared the number of zygotene spermatocytes at day 5. The percentage of zygotene cells in the

37°C-SK49 and 37°C-TM4 groups at day 5 was significantly higher than in the 34°C-SK49 and 34°C-TM4 groups (Figure 2E), while there was no significant difference between the SK49 and the TM4 groups when cultured at the same temperature.

For the SK49 groups, we additionally compared the number of zygotene, pachytene-like, and M-phase cells at 34°C and 37°C after 7 days of culture. Although the percentage of zygotene cells did not differ between 34°C and 37°C, the number of pachytene-like cells in the 37°C-SK49 group was significantly higher than that in the 34°C-SK49 group (Figure 2F). However, the number of M-phase cells at day 7 was significantly higher at 34°C (Figure 2F). In all, only SK49 Sertoli cells were able to support full meiosis, and all further experiments were conducted using SK49 Sertoli cells and at the optimal temperature of 34°C.

Meiotic Homologous Chromosome Synapsis and DNA Double-Strand Break Repair Are Not Completed *In Vitro*

To further characterize *in vitro* meiosis, we stained *in vitro*-formed spermatocytes using antibodies against γH2AX, a marker for DNA double-strand breaks (DSBs) that stains unsynapsed chromosomes and is usually restricted to the XY body during pachytene *in vivo* (Figure S1A) (Mahadevaiah et al., 2001). During the *in vitro* leptotene and zygotene stages, when chromosome synapsis is still proceeding, we observed γH2AX staining throughout the nucleus. However, *in vitro*, this staining did not disappear completely, and clear γH2AX staining remained present on many autosomes during the pachytene-like stage (Figure 3A).

Similarly, although independent of the presence of DSBs (Bellani et al., 2005; Widger et al., 2018), the ataxia telangiectasia and Rad3-related protein (ATR) is known to remain during pachytene only on unsynapsed meiotic homologous chromosomes (Royo et al., 2013). In line with ATR staining for *in vivo* spermatocytes (Figure S1C), we found ATR staining as small dots in the nucleus during the *in vitro* leptotene stage, while it was located at the unsynapsed axial elements of the synaptonemal complex during the *in vitro* zygotene stage. However, like for γH2AX, we observed remaining ATR staining on many homologous chromosome pairs in *in vitro*-generated pachytene-like cells, while ATR staining appeared only on the XY body in pachytene spermatocytes *in vivo* (Figures 3B and S1C).

In vivo, early repair of meiotic DSBs is marked by RAD51 (Moens et al., 2002), which forms small foci during the leptotene and zygotene meiotic stages (Figure S1B). In our *in vitro* system we indeed observed RAD51 foci throughout the nucleus in leptotene cells that accumulated along axial elements of the synaptonemal complex at the zygotene stage. However, at the pachytene-like stage, many RAD51 foci remained present, while *in vivo* RAD51 foci become restricted to the unsynapsed X and Y chromosome XY body in pachytene spermatocytes, indicating suboptimal synapsis or DSB repair during *in vitro* meiosis (Figures 4A and S1B).

Optimal meiotic DSB repair will normally lead to formation of one or two meiotic crossovers per homologous chromosome pair, which, at the pachytene stage, can be marked using antibodies against the MutL homolog 1 protein MLH1 (Moens et al., 2002). These crossovers ensure that the homologous chromosomes remain paired as bivalents until the first meiotic M phase. To investigate crossover formation *in vitro*, we stained for MLH1 in *in vitro*-generated pachytene-like spermatocytes. Although control pachytene spermatocytes, obtained from control intact testis, displayed MLH1 foci as expected, no MLH1 foci were observed in *in vitro*-generated pachytene-like spermatocytes (Figure 4B). Because meiotic crossovers are essential to form bivalents, we followed a karyotyping meiotic spreading protocol at day 8 after spermatogonial differentiation to better visualize the chromosomes of *in vitro*-generated meiotic M phase. In accordance with the absence of MLH1 foci, only M-phase cells with univalents were formed *in vitro* (Figure 4C).

In Vitro-Formed Flower-Shaped Cells Are Most Likely Premature Meiotic M-Phase Cells

A cell type that we observed, but that to our knowledge does not exist *in vivo*, is the flower-shaped meiotic cell. Because all their centromeres are located in the center of the cell (Figure 1D), we investigated whether these might represent some sort of chromosome bouquet formation. Chromosome bouquet formation is the phenomenon of the movement and clustering of telomeres along the nuclear envelope during meiosis in (pre-)zygotene that precedes homologous chromosome pairing (Scherthan et al., 1996). Clustered attachment of the telomeres to the nuclear envelope is characterized by co-localization of the protein SUN1, which is essential for this process (Ding et al., 2007). Because mouse centromeres are located at one of the telomeric ends, they also co-localize with SUN1 in zygotene and pachytene in spermatocytes *in vivo* (Figure S1D). However, in the flower-shaped cells in our culture system, SUN1 did not co-localize with centrally clustered centromeres and these cells did not appear to be meiotic bouquet cells (Figure 5A).

Based on DAPI staining of DNA, the chromatin in the flower-shaped cells appears totally different from a meiotic bouquet. The condensed meiotic chromosomes appear to be in M phase. However, in contrast to the small patches of SYCP3 around the centromeres that we observed in "true" M-phase cells, they displayed long fragmented stretches of SYCP3 along their entire chromosome lengths

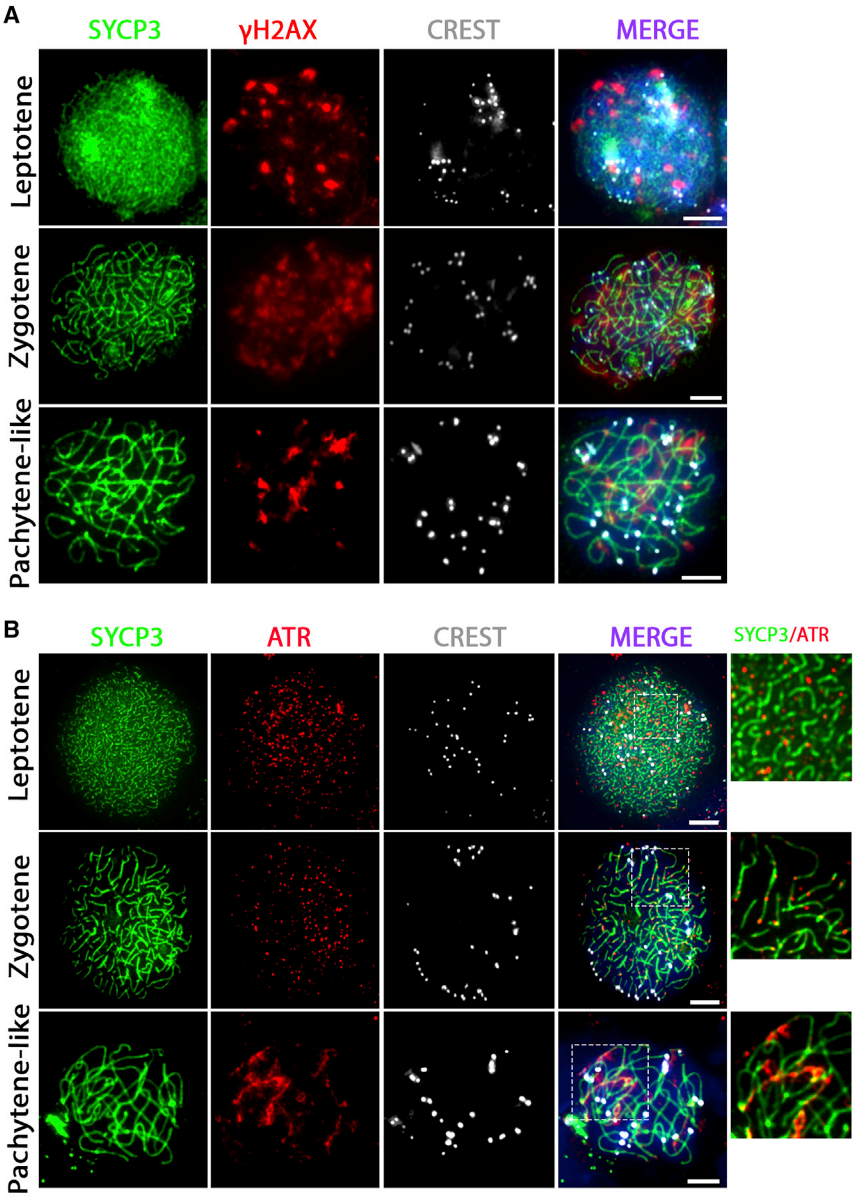

Figure 3. Incomplete Synapsis of the Homologous Chromosome Synapsis during *In Vitro* Meiosis

Unsynapsed homologous chromosomes marked with γH2AX (A) or ATR (B) remain in *in vitro*-generated pachytene-like spermatocytes stained for SYCP3 (green), γH2AX or ATR (red), centromeres (CREST, white), and DNA (DAPI, blue in merged images). Regions inside dashed boxes are shown at higher magnification in the following image. Scale bars, 5 μm.

(Figure 1D). Moreover, they appeared in culture before the appearance of pachytene-like spermatocytes (Figures 1A, 1B, and 2A). It has been shown that isolated spermatocytes can be induced into an M-phase-like state prematurely by okadaic acid (OA) (Tarsounas et al., 1999; Wiltshire et al., 1995). To explore whether the flower cells could be meiotic cells that prematurely entered the first meiotic M-phase (MI), we forced *in vitro*-generated spermatocytes into MI by adding OA to the culture medium at day 9 and compared their morphology with that of the flower-shaped cells. We found that M-phase-like cells could be produced after 4 h of OA treatment. In line with a previous study (Gómez et al., 2016), the distribution of SYCP3 in the OA-induced MI-like cells was similar to that in the flower-shaped cells and formed fragmented stretches along the condensed chromosome arms (Figure 5B). However, in contrast to flower-shaped cells, the centromeres of OA-induced cells were not clustered in the middle of the cells (Figure 5B). Moreover, we did not observe an increased number of flower-shaped cells in response to OA. Unlike the OA-induced MI-like cells, M-phase cells in the *in vitro* differentiation cultures without OA treatment did not display stretches of SYCP3, but only staining at the centromere region (Figure 1D).

To further investigate whether the flower-shaped cells could be premature M-phase spermatocytes, we studied

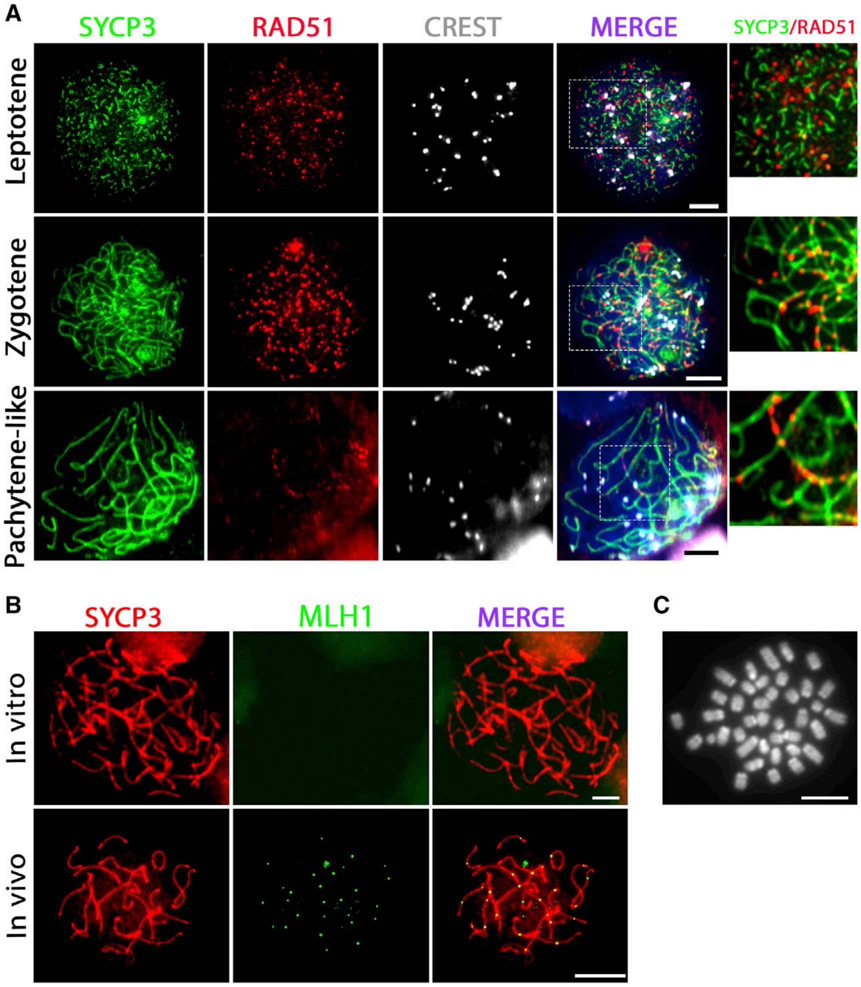

Figure 4. Meiotic DNA Double-Strand Breaks Are Initiated but No Crossovers Are Formed *In Vitro*

(A and B) DSBs marked by RAD51 (A) are initiated in *in vitro*-generated leptotene spermatocytes but are not completely resolved at the pachytene-like stage. Stained for SYCP3 (green), RAD51 (red), centromeres (CREST, white), and DNA (DAPI, blue in merged images). Regions inside dashed boxes are shown at higher magnification in the following image. Meiotic crossovers marked by MLH1 (B) are not formed *in vitro* (top) as compared with *in vivo* (bottom); SYCP3 (red) and MLH1 (green).

(C) Karyotype of *in vitro*-generated meiotic M-phase cells displaying univalents (pairs of sister chromosomes). Scale bars, 5 μm.

in vitro meiotic spindle formation by using an antibody against α-Tubulin (Figure 5C). As a positive control we used undifferentiated mitotically dividing GSCs. In these mitotic M-phase cells no SYCP3 was present on the chromosomes, which were condensed with their centromeres co-localizing with α-Tubulin-fibers. In *in vitro*-generated meiotic M-phase spermatocytes, the α-Tubulin-fibers looked a bit less organized but were nevertheless clearly visible and also ended co-localizing with the centromeres and small patches of SYCP3. In the flower-shaped cells α-Tubulin was mostly present in the center, clustering together with the centromeres. Nevertheless, α-Tubulin-fibers were also clearly visible and appeared very similar to those in *in vitro*-generated meiotic M-phase cells. Hence, the flower-shaped cells appeared very similar to *in vitro*-generated meiotic M-phase cells, with the difference that their centromeres were clustered in the center of the cell. In addition, SYCP3 in the flower-shaped cells was not restricted to the centromeric sites, similar to the meiotic cells that were prematurely forced into M phase by OA treatment.

In line with an M-phase phenotype, the flower-shaped cells often appear in pairs. In contrast to single flower cells, each of the cells within these pairs contains about half the centromere dots (Figure 5D). However, in contrast to a well-organized division, these are not exactly evenly divided (Figure 5E). All things considered, we conclude that the flower-shaped cells are most likely *in vitro*-generated meiotic cells that prematurely entered the meiotic M phase, causing an uneven division of the meiotic chromosomes.

Inefficient Generation of Round Spermatid-like Cells

Occasionally, but very rarely, we observed the generation of round spermatid-like cells that also formed an acrosome-like structure that stained positive for PNA (Figure 1D). To further analyze the appearance of these cells, generally after day 7, we harvested cells at day 8, followed by fluorescence-activated cell sorting (FACS) for DNA content. As

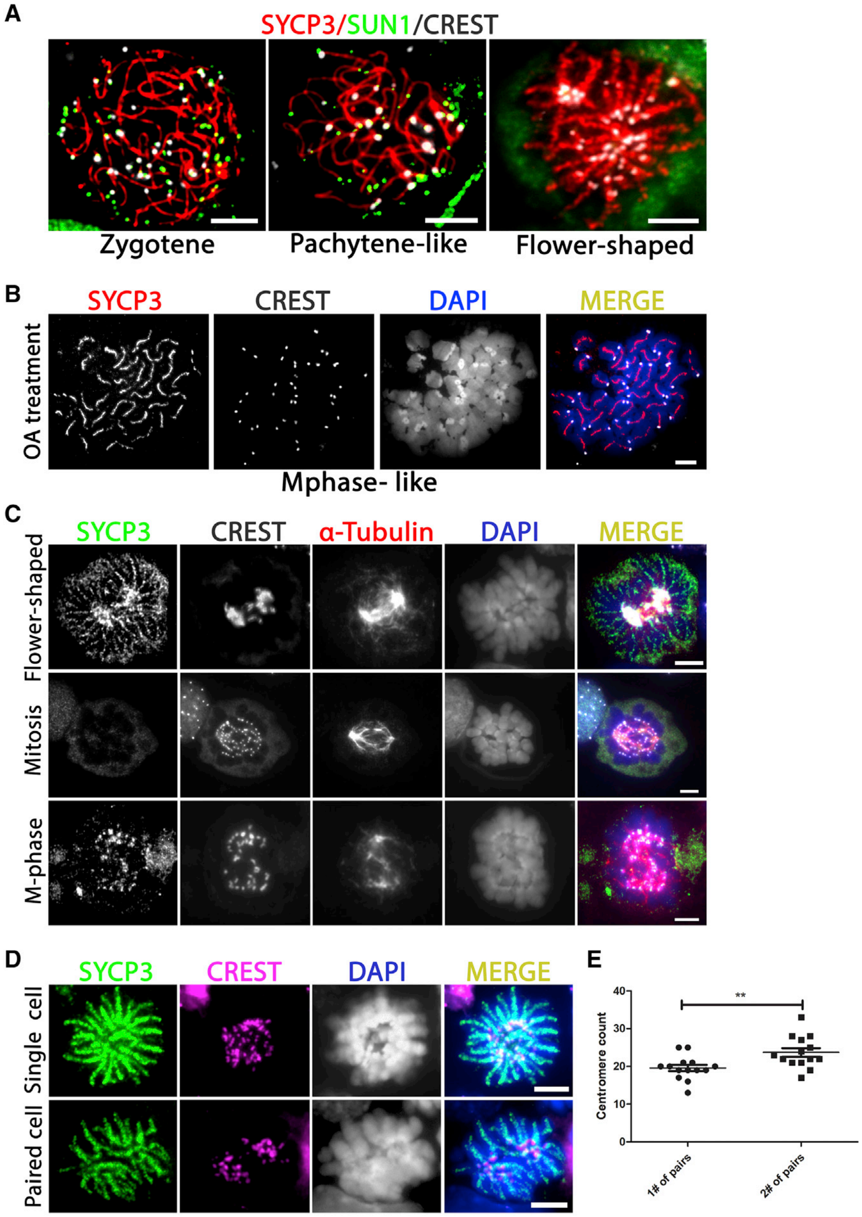

Figure 5. *In Vitro*-Formed Flower-Shaped Cells Resemble Premature M-Phase Spermatocytes

(A) Unlike bouquet-stage meiotic cells, the clustered centromeres in the flower-shaped cells (CREST, white) do not co-localize with the protein SUN1 (green).

(B) Like flower-shaped cells, okadaic acid (OA)-induced M-phase cells also form stretches of SYCP3 along the chromosome arms; SYCP3 (red in merged), centromeres (CREST, white in merged), and DNA (DAPI, blue in merged).

(C) As in mitotic and meiotic M-phase cells, α-Tubulin (red) in flower-shaped cells forms stretches that end by co-localizing with the centromeres (CREST, white in merged; SYCP3, green).

(D) The flower-shaped cells frequently appear in pairs of recently divided cells containing about half of the chromosomes; SYCP3 (green), centromeres (CREST, red), and DNA (DAPI, white/blue in merged).

(E) Pairs of flower-shaped cells display an uneven distribution of chromosomes (centromeres) between the first (1#) and the second (2#) cell within a pair; $^{**}p < 0.01$. Scale bars, 5 μm.

negative control for a possible haploid peak (1C) we used undifferentiated GSCs. For positive control we used mouse testicular cells. The experimental group generated a rounded peak in the FACS analyses that may include 1C-haploid cells (Figure 6A). In line with the microscopy results, we also found a clear 4C region in the experimental group. However, the peak of this region appeared moved to the left, which may be caused by premature meiotic entry and M phase before completion of the meiotic S phase. Subsequently, we sorted cells present in the putative "1C region" and made cytospin microscope slides to stain for the acrosome marker PNA. Of approximately 500 sorted cells only 10 appeared positive for PNA (Figure 6B). As positive control we used mouse testis sections, in which a clear PNA-positive acrosome cap was visible on the round spermatids. Hence, spermiogenesis seems to occur *in vitro*, but with very low efficiency and resulting in mostly spermatid-like cells with irregular chromosomal content.

DISCUSSION

In our study, we investigated whether spermatogenesis can be mimicked *in vitro* in a cell culture by starting with freshly

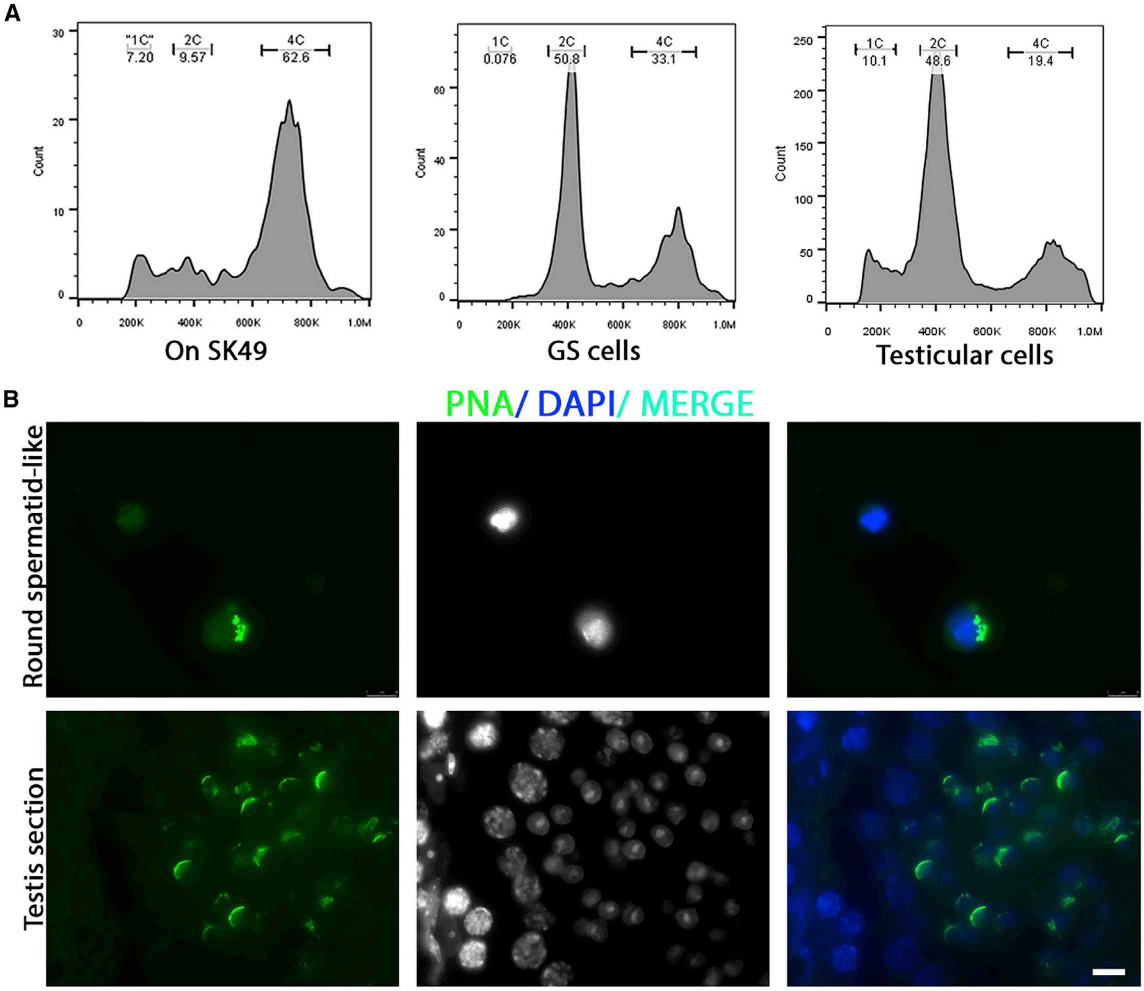

Figure 6. Inefficient Generation of Round Spermatid-like Cells *In Vitro*
(A) FACS analysis on DNA content of GSCs cultured in an *in vitro* meiosis system at day 8 compared with undifferentiated GSCs and mouse testicular cells.
(B) Acrosome staining (PNA, green) of 1C-sorted spermatid-like cells (top) compared with spermatids in normal mouse testis sections (bottom). Scale bar, 8 μm.

isolated SSCs without co-culture with juvenile gonadal tissue. To this end, we used primary isolated mouse spermatogonia, referred to as male GSCs (Kanatsu-Shinohara et al., 2003). Using a three-step culture system, including germ cell culture on a feeder layer of Sertoli cells, we observed initiation and progression of meiosis *in vitro*. However, the first meiotic prophase proceeded faster than *in vivo*, with many unsynapsed areas between homologous chromosomes at the pachytene-like stage, and no visible meiotic crossovers. This led to formation of meiotic M-phase cells with univalents (pairs of sister chromosomes) at a stage during which bivalents (pairs of homologous chromosomes) should be present. Spermatid-like cells, although stained for the acrosome marker PNA, were formed at only very low efficiency. In addition, before the appearance of pachytene-like spermatocytes, the meiotic M phase was often initiated prematurely *in vitro*, visible as flower-shaped cells that, to our knowledge, have not been described previously.

In line with other studies that used FACS for the analysis and isolation of *in vitro*-formed round spermatids (Geijsen et al., 2004; Nayernia et al., 2006; Sato et al., 2011a; Zhou et al., 2016), we used FACS to determine the DNA content of *in vitro*-generated meiotic and spermatid-like cells. The aberrant divisions of these flower-shaped cells may explain the round, broader 1C peak in our FACS analysis. If this peak really represented haploid spermatids, one would expect a high number of PNA-positive cells. However, spermatid-like PNA-positive cells occurred only extremely rarely in our culture system. Due to their low numbers,

these cells could not be isolated for further analysis, but, considering the absence of meiotic crossovers in our *in vitro* system, we would expect that most of them would be aneuploid. In addition, premature M phase of the flower-shaped cells may also explain the lower DNA content of the 4C peak within the 4C region. This could be caused by meiotic entry before completion of the meiotic S phase after spermatogonial differentiation in combination with the low abundance of mitotically active cells at day 8, which affects the mitotic S- and G2-phase peaks.

As published for the culture of neonatal mouse testis tissue fragments (Gohbara et al., 2010; Sato et al., 2011a), the optimal temperature for *in vitro* spermatogenesis appears to be 34°C, rather than the 37°C normal for most cell culture systems. Indeed, it is also known that scrotal temperature is usually several degrees lower than the core body temperature (Waites and Setchell, 1964; Werdelin and Nilsonne, 1999). However, lowering the temperature to 34°C did not prevent the observed block in *in vitro* meiotic divisions and haploid spermatid production. Moreover, in our experiments comparing two Sertoli cell lines and in testis tissue fragment cultures (Portela et al., 2019b), the somatic background seems to have a determinative effect on the germ cell developmental potential *in vitro*. Apart from the somatic supporting cells, the genetic background of the SSCs also determines how well they can be maintained *in vitro* (Kanatsu-Shinohara et al., 2003; Portela et al., 2019b). Should any form of *in vitro* gametogenesis be considered as treatment for human infertility, the molecular mechanism that underlies these differences should be investigated. These mechanisms are also highly relevant for any fertility treatment or preservation/restoration strategy that depends on culture of human SSCs. Without further research in this area, varying chances of success will be patient specific and not understood.

Strikingly, *in vitro* spermatogenesis seems more difficult to achieve when using SSCs as a starting point than when starting with pluripotent stem cells. In 2016, Zhou et al. used ESCs to generate PGCLCs that were induced to undergo meiosis while being co-cultured with juvenile testicular cells (Zhou et al., 2016). Another study used a *Stra8-EGFP* transgenic mouse line to isolate fluorescent spermatogonia and generate maGSCs that differentiated to spermatids via the ESC-like pathway (Nolte et al., 2010). Importantly, in these systems, PGCLCs or ESC-like cells are directly induced to enter meiosis, skipping the SSC (undifferentiated spermatogonia) and differentiation (differentiating spermatogonia) stages that normally occur during post-pubertal spermatogenesis *in vivo* (de Rooij and Russell, 2000). Our *in vitro* spermatogenesis system starts with undifferentiated spermatogonia that, as *in vivo*, are first induced to become differentiating spermatogonia (Zheng et al., 2018) before induction of meiosis, giving them another starting point for meiosis induction compared with pluripotent stem cell-derived PGCLCs.

In contrast to the mitotic prophase, which takes only minutes, the mouse meiotic prophase normally takes about 2 weeks (Oakberg, 1956). When meiosis is initiated, a meiotic cell-cycle program including the proteins MEIOC and YTDC2 suppresses mitotic cell-cycle factors, such as Cyclin A2, that are present in spermatogonia (Soh et al., 2017). In our culture system, M-phase meiotic cells appear already after 7 days, which could thus be due to mitotic cell-cycle factors still present during the meiotic prophase *in vitro*. In addition, we observed flower-shaped cells that appeared to have entered the meiotic M phase prematurely, before the appearance of pachytene-like cells. Comparable to these flower-shaped cells, *Meioc*$^{-/-}$ mice also prematurely initiate the meiotic M phase, which also leads to meiotic M-phase spermatocytes that form only univalent metaphases (Soh et al., 2017). However, unlike our cells with flower-shaped chromatin arrangements, *Meioc*$^{-/-}$ spermatocytes do not cluster their centromeres in the middle of the cell.

Other meiotic problems we observed *in vitro* could also be due to a shortened prophase time, for instance, the lower efficiency of synapsis between the homologous chromosomes. However, when starting with PGCLCs, the meiotic prophase could be finished within 1 week without reported problems with chromosome synapsis, at which 64% of the cultured cells were at the pachytene stage and 50% cells had entered the diplotene stage (Zhou et al., 2016). Apparently, the starting cell type has more influence on the completion of synapsis than the shortened prophase. On the other hand, when PGCLCs were co-cultured with embryonic ovary tissue to generate oocytes, about 55% of pachytene-like oocytes displayed unsynapsed homologous chromosomes *in vitro*, compared with only 5% *in vivo* (Hikabe et al., 2016). In another study, PGCLCs were induced by BMP2 and RA without the use of gonadal somatic cells, leading to *in vitro* generation of about 12% pachytene-like oocytes (Miyauchi et al., 2017). When PGCLCs were transfected to overexpress ZGLP1, a determinant for the oogenic fate in mice, up to 14% of them proceeded to the pachytene-like stage (Nagaoka et al., 2020). In contrast, *in vivo*, almost all oocytes reach the pachytene stage (Borum, 1961). Hence, inefficient synapsis of the homologous chromosomes during the pachytene stage seems characteristic of *in vitro* meiosis.

Apart from chromosome synapsis, the *in vitro*-generated spermatocytes also appear to have problems with the repair of meiotic DNA DSBs. *In vivo*, the repair of these DSBs both initiates and requires normal meiotic chromosome synapsis. Moreover, proper DSB repair leads to the formation of at least one meiotic crossover per homologous chromosome pair (de Massy, 2013). These meiotic crossovers

physically link the homologous chromosome pairs at the start of the first meiotic M phase, leading to the typical bivalent shape of these chromosome pairs. Although early DSB repair is initiated in our *in vitro* system, meiotic crossovers are not formed, perhaps because synapsis is incomplete. As a consequence, the homologous chromosomes are not paired in bivalents during the first meiotic M phase *in vitro*, but instead appear as univalent pairs of sister chromatids.

Both in mouse (de Rooij and de Boer, 2003; Hamer et al., 2008) and in human (Jan et al., 2018), failure of meiotic DSB repair or chromosome synapsis leads to meiotic prophase arrest and subsequent spermatocyte apoptosis. Meiotic arrest prevents the occurrence of univalent chromosomes at the first meiotic M phase, which otherwise could lead to improper chromosome alignment during the first meiotic division and subsequent formation of aneuploid gametes. Importantly, meiotic checkpoints have so far not been investigated for *in vitro* gametogenesis. We now show that, despite the presence of unsynapsed chromosomal regions and lack of meiotic crossovers, *in vitro*-generated spermatocytes still proceed to the meiotic M phase and thus lack a fully functional meiotic prophase checkpoint. Before *in vitro* gametogenesis can be considered for clinical use, regardless of whether pluripotent stem cells or spermatogonia are used as the starting cell type, meiotic checkpoint functionality should be investigated because meiotic checkpoints prevent certain genomic abnormalities from being passed on to the offspring. We therefore argue that, in addition to chromosome content and organization, proper meiotic recombination, and viable euploid offspring, as outlined by Handel and colleagues in 2014 (Handel et al., 2014), analysis of meiotic checkpoints should be added to the "gold standards" of *in vitro*-derived gametes.

EXPERIMENTAL PROCEDURES

Animals

Neonatal (4–5 dpp) DBA/2J male mice were used for isolation of primary spermatogonia (male GSCs). All animal procedures were in accordance with and approved by the animal ethics committee of the Amsterdam UMC, Academic Medical Center, University of Amsterdam.

In Vitro Meiosis of GSCs

GSCs, cultured on a feeder layer of mitomycin-inactivated Sertoli cells, were induced to undergo meiosis by StemPro-34 SFM medium containing StemPro-34 supplement, 10% KnockOut serum replacement (KSR), RA, recombinant mouse BMP4 protein, and recombinant mouse Activin A protein; this was followed by StemPro-34 SFM medium containing StemPro-34 supplement, 10% KSR, BPE, FSH, and testosterone. For details see Supplemental Experimental Procedures.

Cytology

Meiotic spread preparations were prepared as previously described (Jan et al., 2018). Alternatively, all cultured cells, including non-adherent cells, were spread on the slides using a cytospin (CELLSPIN, 521-1990, VWR). Details of the cytospin procedure can be found in the Supplemental Experimental Procedures.

To visualize the progress of spermatogenesis *in vitro*, cytospin slides were used. After the cells were cytospun, they (cytospins) were fixed, permeabilized, and blocked as previously described (Zheng et al., 2017), followed by overnight incubation at 4°C with primary antibodies (Table S1). For negative controls, primary antibodies were replaced with complementary immunoglobulin G. On the next day, the cytospins were washed and incubated with the corresponding host-specific secondary antibodies (Table S1) and counterstained with DAPI. The cytospins were mounted with Prolong Gold anti-fade mountant (Thermo Fisher Scientific) and later visualized using a Leica DM5000B microscope.

Images were analyzed using Leica Application Suite X and ImageJ version Java 1.8.0_77. The figures were constructed using Adobe Photoshop CS5 version 13.0.1 and Adobe illustrator version CS6.

For OA-induced generation of M-phase-like cells, karyotyping, and flow cytometry, see Supplemental Experimental Procedures.

Statistics

For quantification of meiotic progression of the early time points (0 and 3 days), at least 100 cells of each group were assessed, leading to a total of at least 300 cells in each group of three independent experiments, excluding Sertoli cells and MEFs based on nuclear size. At later time points (5, 7, and 9 days), because the number of meiotic cells was decreasing, at least 70 cells of each group were assessed, leading to a total of at least 210 cells in each group of three independent experiments. Differences among groups were assessed using one-way ANOVA followed by a least significant difference test. Data are presented as the mean ± SEM of three independent experiments (n = 3).

SUPPLEMENTAL INFORMATION

Supplemental Information can be found online at https://doi.org/10.1016/j.stemcr.2020.10.006.

AUTHOR CONTRIBUTIONS

Q.J.L., A.M.M.v.P., and G.H. designed the experiments. Q.J.L., X.L., and J.E. performed the experiments. Q.J.L. and G.H. analyzed the data. Q.J.L., S.C.S.L., A.M.M.v.P., and G.H. wrote the manuscript.

ACKNOWLEDGMENTS

We thank Manfred Alsheimer for useful discussion and for kindly providing the antibody against SUN1. We thank Callista Mulder and Zheng Yi for assistance with the isolation of primary GSCs. Q.L. is the recipient of a China Scholarship Council grant (201706300107).

Received: May 26, 2020
Revised: October 13, 2020
Accepted: October 14, 2020
Published: November 10, 2020

REFERENCES

Bellani, M.A., Romanienko, P.J., Cairatti, D.A., and Camerini-Otero, R.D. (2005). SPO11 is required for sex-body formation, and Spo11 heterozygosity rescues the prophase arrest of Atm-/- spermatocytes. J. Cell Sci. *118*, 3233–3245.

Bisig, C.G., Guiraldelli, M.F., Kouznetsova, A., Scherthan, H., Höög, C., Dawson, D.S., and Pezza, R.J. (2012). Synaptonemal complex components persist at centromeres and are required for homologous centromere pairing in mouse spermatocytes. PLoS Genet. *8*, e1002701.

Borum, K. (1961). Oogenesis in the mouse: a study of the meiotic prophase. Exp. Cell Res. *24*, 495–507.

Dann, C.T., Alvarado, A.L., Molyneux, L.A., Denard, B.S., Garbers, D.L., and Porteus, M.H. (2008). Spermatogonial stem cell self-renewal requires OCT4, a factor downregulated during retinoic acid-induced differentiation. Stem Cells *26*, 2928–2937.

De Gendt, K., Swinnen, J.V., Saunders, P.T.K., Schoonjans, L., Dewerchin, M., Devos, A., Tan, K., Atanassova, N., Claessens, F., Lécureuil, C., et al. (2004). A Sertoli cell-selective knockout of the androgen receptor causes spermatogenic arrest in meiosis. Proc. Natl. Acad. Sci. U S A *101*, 1327–1332.

de Massy, B. (2013). Initiation of meiotic recombination: how and where? Conservation and specificities among eukaryotes. Annu. Rev. Genet. *47*, 563–599.

de Rooij, D.G., and de Boer, P. (2003). Specific arrests of spermatogenesis in genetically modified and mutant mice. Cytogenet. Genome Res. *103*, 267–276.

de Rooij, D.G., and Russell, L.D. (2000). All you wanted to know about spermatogonia but were afraid to ask. J. Androl. *21*, 776–798.

Ding, X., Xu, R., Yu, J., Xu, T., Zhuang, Y., and Han, M. (2007). SUN1 is required for telomere attachment to nuclear envelope and gametogenesis in mice. Dev. Cell *12*, 863–872.

Easley IV, C.A., Phillips, B.T., McGuire, M.M., Barringer, J.M., Valli, H., Hermann, B.P., Simerly, C.R., Rajkovic, A., Miki, T., and Orwig, K.E. (2012). Direct differentiation of human pluripotent stem cells into haploid spermatogenic cells. Cell Rep. *2*, 440–446.

Eguizabal, C., Montserrat, N., Vassena, R., Barragan, M., Garreta, E., Garcia-Quevedo, L., Vidal, F., Giorgetti, A., Veiga, A., and Belmonte, J.I. (2011). Complete meiosis from human induced pluripotent stem cells. Stem Cells *29*, 1186–1195.

Fayomi, A.P., Peters, K., Sukhwani, M., Valli-Pulaski, H., Shetty, G., Meistrich, M.L., Houser, L., Robertson, N., Roberts, V., and Ramsey, C. (2019). Autologous grafting of cryopreserved prepubertal rhesus testis produces sperm and offspring. Science *363*, 1314–1319.

Feng, L.-X., Chen, Y., Dettin, L., Pera, R.A.R., Herr, J.C., Goldberg, E., and Dym, M. (2002). Generation and in vitro differentiation of a spermatogonial cell line. Science *297*, 392–395.

Geijsen, N., Horoschak, M., Kim, K., Gribnau, J., Eggan, K., and Daley, G.Q. (2004). Derivation of embryonic germ cells and male gametes from embryonic stem cells. Nature *427*, 148.

Gohbara, A., Katagiri, K., Sato, T., Kubota, Y., Kagechika, H., Araki, Y., Araki, Y., and Ogawa, T. (2010). In vitro murine spermatogenesis in an organ culture system. Biol. Reprod. *83*, 261–267.

Gómez, R., Felipe-Medina, N., Ruiz-Torres, M., Berenguer, I., Viera, A., Pérez, S., Barbero, J.L., Llano, E., Fukuda, T., and Alsheimer, M. (2016). Sororin loads to the synaptonemal complex central region independently of meiotic cohesin complexes. EMBO Rep. *17*, 695–707.

Hamada, A.J., Esteves, S.C., and Agarwal, A. (2013). A comprehensive review of genetics and genetic testing in azoospermia. Clinics *68*, 39–60.

Hamer, G., Novak, I., Kouznetsova, A., and Höög, C. (2008). Disruption of pairing and synapsis of chromosomes causes stage-specific apoptosis of male meiotic cells. Theriogenology *69*, 333–339.

Handel, M.A., Eppig, J.J., and Schimenti, J.C. (2014). Applying "gold standards" to in-vitro-derived germ cells. Cell *157*, 1257–1261.

Hayashi, K., Ohta, H., Kurimoto, K., Aramaki, S., and Saitou, M. (2011). Reconstitution of the mouse germ cell specification pathway in culture by pluripotent stem cells. Cell *146*, 519–532.

Hendriks, S., Dancet, E.A., van Pelt, A.M., Hamer, G., and Repping, S. (2015b). Artificial gametes: a systematic review of biological progress towards clinical application. Hum. Reprod. Update *21*, 285–296.

Hendriks, S., Dondorp, W., de Wert, G., Hamer, G., Repping, S., and Dancet, E.A. (2015a). Potential consequences of clinical application of artificial gametes: a systematic review of stakeholder views. Hum. Reprod. Update *21*, 297–309.

Hikabe, O., Hamazaki, N., Nagamatsu, G., Obata, Y., Hirao, Y., Hamada, N., Shimamoto, S., Imamura, T., Nakashima, K., Saitou, M., et al. (2016). Reconstitution in vitro of the entire cycle of the mouse female germ line. Nature *539*, 299–303.

Ishikura, Y., Yabuta, Y., Ohta, H., Hayashi, K., Nakamura, T., Okamoto, I., Yamamoto, T., Kurimoto, K., Shirane, K., and Sasaki, H. (2016). In vitro derivation and propagation of spermatogonial stem cell activity from mouse pluripotent stem cells. Cell Rep. *17*, 2789–2804.

Jan, S.Z., Jongejan, A., Korver, C.M., van Daalen, S.K., van Pelt, A.M., Repping, S., and Hamer, G. (2018). Distinct prophase arrest mechanisms in human male meiosis. Development *145*, dev160614.

Kanatsu-Shinohara, M., Ogonuki, N., Inoue, K., Miki, H., Ogura, A., Toyokuni, S., and Shinohara, T. (2003). Long-term proliferation in culture and germline transmission of mouse male germline stem cells. Biol. Reprod. *69*, 612–616.

Kojima, Y., Sasaki, K., Yokobayashi, S., Sakai, Y., Nakamura, T., Yabuta, Y., Nakaki, F., Nagaoka, S., Woltjen, K., and Hotta, A. (2017). Evolutionarily distinctive transcriptional and signaling programs drive human germ cell lineage specification from pluripotent stem cells. Cell Stem Cell *21*, 517–532.e5.

Krausz, C. (2011). Male infertility: pathogenesis and clinical diagnosis. Best Pract. Res. Clin. Endocrinol. Metab. *25*, 271–285.

Kumar, N., and Singh, A.K. (2015). Trends of male factor infertility, an important cause of infertility: a review of literature. J. Hum. Reprod. Sci. *8*, 191.

Li, N., Ma, W., Shen, Q., Zhang, M., Du, Z., Wu, C., Niu, B., Liu, W., and Hua, J. (2019). Reconstitution of male germline cell specification from mouse embryonic stem cells using defined factors in vitro. Cell Death Differ. *26*, 2115–2124.

Mahadevaiah, S.K., Turner, J.M., Baudat, F., Rogakou, E.P., de Boer, P., Blanco-Rodríguez, J., Jasin, M., Keeney, S., Bonner, W.M., and Burgoyne, P.S. (2001). Recombinational DNA double-strand breaks in mice precede synapsis. Nat. Genet. *27*, 271.

Matfier, J.P. (1980). Establishment and characterization of two distinct mouse testicular epithelial cell lines. Biol. Reprod. *23*, 243–252.

Medrano, J.V., Vilanova-Pérez, T., Fornés-Ferrer, V., Navarro-Gomezlechon, A., Martínez-Triguero, M.L., García, S., Gómez-Chacón, J., Povo, I., Pellicer, A., and Andrés, M.M. (2018). Influence of temperature, serum, and gonadotropin supplementation in short-and long-term organotypic culture of human immature testicular tissue. Fertil. Sterility *110*, 1045–1057.e3.

Miyauchi, H., Ohta, H., Nagaoka, S., Nakaki, F., Sasaki, K., Hayashi, K., Yabuta, Y., Nakamura, T., Yamamoto, T., and Saitou, M. (2017). Bone morphogenetic protein and retinoic acid synergistically specify female germ-cell fate in mice. EMBO J. *36*, 3100–3119.

Moens, P.B., Kolas, N.K., Tarsounas, M., Marcon, E., Cohen, P.E., and Spyropoulos, B. (2002). The time course and chromosomal localization of recombination-related proteins at meiosis in the mouse are compatible with models that can resolve the early DNA-DNA interactions without reciprocal recombination. J. Cell Sci. *115*, 1611–1622.

Mulder, C.L., Catsburg, L.A.E., Zheng, Y., de Winter-Korver, C.M., van Daalen, S.K.M., van Wely, M., Pals, S., Repping, S., and van Pelt, A.M.M. (2017). Long-term health in recipients of transplanted in vitro propagated spermatogonial stem cells. Hum. Reprod. *33*, 81–90.

Mulder, C.L., Zheng, Y., Jan, S.Z., Struijk, R.B., Repping, S., Hamer, G., and van Pelt, A.M. (2016). Spermatogonial stem cell autotransplantation and germline genomic editing: a future cure for spermatogenic failure and prevention of transmission of genomic diseases. Hum. Reprod. Update *22*, 561–573.

Nagaoka, S.I., Nakaki, F., Miyauchi, H., Nosaka, Y., Ohta, H., Yabuta, Y., Kurimoto, K., Hayashi, K., Nakamura, T., and Yamamoto, T. (2020). ZGLP1 is a determinant for the oogenic fate in mice. Science *367*, eaaw4115.

Nayernia, K., Nolte, J., Michelmann, H.W., Lee, J.H., Rathsack, K., Drusenheimer, N., Dev, A., Wulf, G., Ehrmann, I.E., and Elliott, D.J. (2006). In vitro-differentiated embryonic stem cells give rise to male gametes that can generate offspring mice. Dev. Cell *11*, 125–132.

Nolte, J., Michelmann, H.W., Wolf, M., Wulf, G., Nayernia, K., Meinhardt, A., Zechner, U., and Engel, W. (2010). PSCDGs of mouse multipotent adult germline stem cells can enter and progress through meiosis to form haploid male germ cells in vitro. Differentiation *80*, 184–194.

Oakberg, E.F. (1956). Duration of spermatogenesis in the mouse and timing of stages of the cycle of the seminiferous epithelium. Am. J. Anat. *99*, 507–516.

Portela, J.M., de Winter-Korver, C.M., van Daalen, S.K., Meißner, A., de Melker, A.A., Repping, S., and van Pelt, A.M. (2019a). Assessment of fresh and cryopreserved testicular tissues from (pre) pubertal boys during organ culture as a strategy for in vitro spermatogenesis. Hum. Reprod. *34*, 2443–2455.

Portela, J.M., Mulder, C.L., van Daalen, S.K., de Winter-Korver, C.M., Stukenborg, J.-B., Repping, S., and van Pelt, A.M. (2019b). Strains matter: success of murine in vitro spermatogenesis is dependent on genetic background. Dev. Biol. *456*, 25–30.

Ramaswamy, S., and Weinbauer, G.F. (2014). Endocrine control of spermatogenesis: role of FSH and LH/testosterone. Spermatogenesis *4*, e996025.

Royo, H., Prosser, H., Ruzankina, Y., Mahadevaiah, S.K., Cloutier, J.M., Baumann, M., Fukuda, T., Höög, C., Tóth, A., de Rooij, D.G., et al. (2013). ATR acts stage specifically to regulate multiple aspects of mammalian meiotic silencing. Genes Development *27*, 1484–1494.

Sadri-Ardekani, H., Akhondi, M.A., van der Veen, F., Repping, S., and van Pelt, A.M. (2011). In vitro propagation of human prepubertal spermatogonial stem cells. JAMA *305*, 2416–2418.

Sadri-Ardekani, H., Mizrak, S.C., van Daalen, S.K., Korver, C.M., Roepers-Gajadien, H.L., Koruji, M., Hovingh, S., de Reijke, T.M., de la Rosette, J.J., and van der Veen, F. (2009). Propagation of human spermatogonial stem cells in vitro. JAMA *302*, 2127–2134.

Sasaki, K., Yokobayashi, S., Nakamura, T., Okamoto, I., Yabuta, Y., Kurimoto, K., Ohta, H., Moritoki, Y., Iwatani, C., and Tsuchiya, H. (2015). Robust in vitro induction of human germ cell fate from pluripotent stem cells. Cell Stem Cell *17*, 178–194.

Sato, T., Katagiri, K., Gohbara, A., Inoue, K., Ogonuki, N., Ogura, A., Kubota, Y., and Ogawa, T. (2011a). In vitro production of functional sperm in cultured neonatal mouse testes. Nature *471*, 504–507.

Sato, T., Katagiri, K., Yokonishi, T., Kubota, Y., Inoue, K., Ogonuki, N., Matoba, S., Ogura, A., and Ogawa, T. (2011b). In vitro production of fertile sperm from murine spermatogonial stem cell lines. Nat. Commun. *2*, 1–7.

Scherthan, H., Weich, S., Schwegler, H., Heyting, C., Härle, M., and Cremer, T. (1996). Centromere and telomere movements during early meiotic prophase of mouse and man are associated with the onset of chromosome pairing. J. Cell Biol. *134*, 1109–1125.

Soh, Y.S., Mikedis, M.M., Kojima, M., Godfrey, A.K., de Rooij, D.G., and Page, D.C. (2017). Meioc maintains an extended meiotic prophase I in mice. PLoS Genet. *13*, e1006704.

Sun, M., Yuan, Q., Niu, M., Wang, H., Wen, L., Yao, C., Hou, J., Chen, Z., Fu, H., Zhou, F., et al. (2018). Efficient generation of functional haploid spermatids from human germline stem cells by three-dimensional-induced system. Cell Death Differ. *25*, 749–766.

Tarsounas, M., Pearlman, R.E., and Moens, P.B. (1999). Meiotic activation of rat pachytene spermatocytes with okadaic acid: the

behaviour of synaptonemal complex components SYN1/SCP1 and COR1/SCP3. J. Cell Sci. *112*, 423–434.

Waites, G., and Setchell, B. (1964). Effect of local heating on blood flow and metabolism in the testis of the conscious ram. Reproduction *8*, 339–349.

Walther, N., Jansen, M., Ergün, S., Kascheike, B., and Ivell, R. (1996). Sertoli cell lines established fromH-2Kb-tsA58 transgenic mice differentially regulate the expression of cell-specific genes. Exp. Cell Res. *225*, 411–421.

Wang, S., Wang, X.X., Ma, L.F., Lin, X.W., Zhang, D.Q., Li, Z., Wu, Y.J., Zheng, C.W., Feng, X., Liao, S.Y., et al. (2016). Retinoic acid is sufficient for the in vitro induction of mouse spermatocytes. Stem Cell Reports *7*, 80–94.

Werdelin, L., and Nilsonne, Å. (1999). The evolution of the scrotum and testicular descent in mammals: a phylogenetic view. J. Theor. Biol. *196*, 61–72.

Widger, A., Mahadevaiah, S.K., Lange, J., ElInati, E., Zohren, J., Hirota, T., Pacheco, S., Maldonado-Linares, A., Stanzione, M., and Ojarikre, O. (2018). ATR is a multifunctional regulator of male mouse meiosis. Nat. Commun. *9*, 2621.

Wiltshire, T., Park, C., Caldwell, K.A., and Handel, M.A. (1995). Induced premature G2/M-phase transition in pachytene spermatocytes includes events unique to meiosis. Dev. Biol. *169*, 557–567.

Yokonishi, T., Sato, T., Komeya, M., Katagiri, K., Kubota, Y., Nakabayashi, K., Hata, K., Inoue, K., Ogonuki, N., and Ogura, A. (2014). Offspring production with sperm grown in vitro from cryopreserved testis tissues. Nat. Commun. *5*, 1–6.

Zheng, Y., Jongejan, A., Mulder, C.L., Mastenbroek, S., Repping, S., Wang, Y., Li, J., and Hamer, G. (2017). Trivial role for NSMCE2 during in vitro proliferation and differentiation of male germline stem cells. Reproduction *154*, 81–95.

Zheng, Y., Lei, Q., Jongejan, A., Mulder, C.L., van Daalen, S.K., Mastenbroek, S., Hwang, G., Jordan, P.W., Repping, S., and Hamer, G. (2018). The influence of retinoic acid-induced differentiation on the radiation response of male germline stem cells. DNA Repair *70*, 55–66.

Zhou, Q., Wang, M., Yuan, Y., Wang, X., Fu, R., Wan, H., Xie, M., Liu, M., Guo, X., and Zheng, Y.J.C.s.c. (2016). Complete meiosis from embryonic stem cell-derived germ cells in vitro. Cell Stem Cell *18*, 330–340.

Stem Cell Reports, Volume *15*

Supplemental Information

In Vitro Meiosis of Male Germline Stem Cells

Qijing Lei, Xin Lai, Jitske Eliveld, Susana M. Chuva de Sousa Lopes, Ans M.M. van Pelt, and Geert Hamer

Supplemental Information

In vitro meiosis of male germline stem cells

Qijing Lei[1], Xin Lai[1], Jitske Eliveld[1], Susana M. Chuva de Sousa Lopes[2], Ans M.M. van Pelt[1], Geert Hamer[1,*]

[1]Center for Reproductive Medicine, Reproductive Biology Laboratory, Amsterdam Reproduction and Development Research Institute, Amsterdam UMC, Location AMC, University of Amsterdam, 1105AZ, Amsterdam, the Netherlands

[2]Department of Anatomy and Embryology, Leiden University Medical Center, 2333 ZC, Leiden, Netherlands

*Corresponding address. Center for Reproductive Medicine, Reproductive Biology Laboratory, Amsterdam UMC, Location AMC, Meibergdreef 9, 1105 AZ, Amsterdam, The Netherlands. E-mail: g.hamer@amsterdamumc.nl

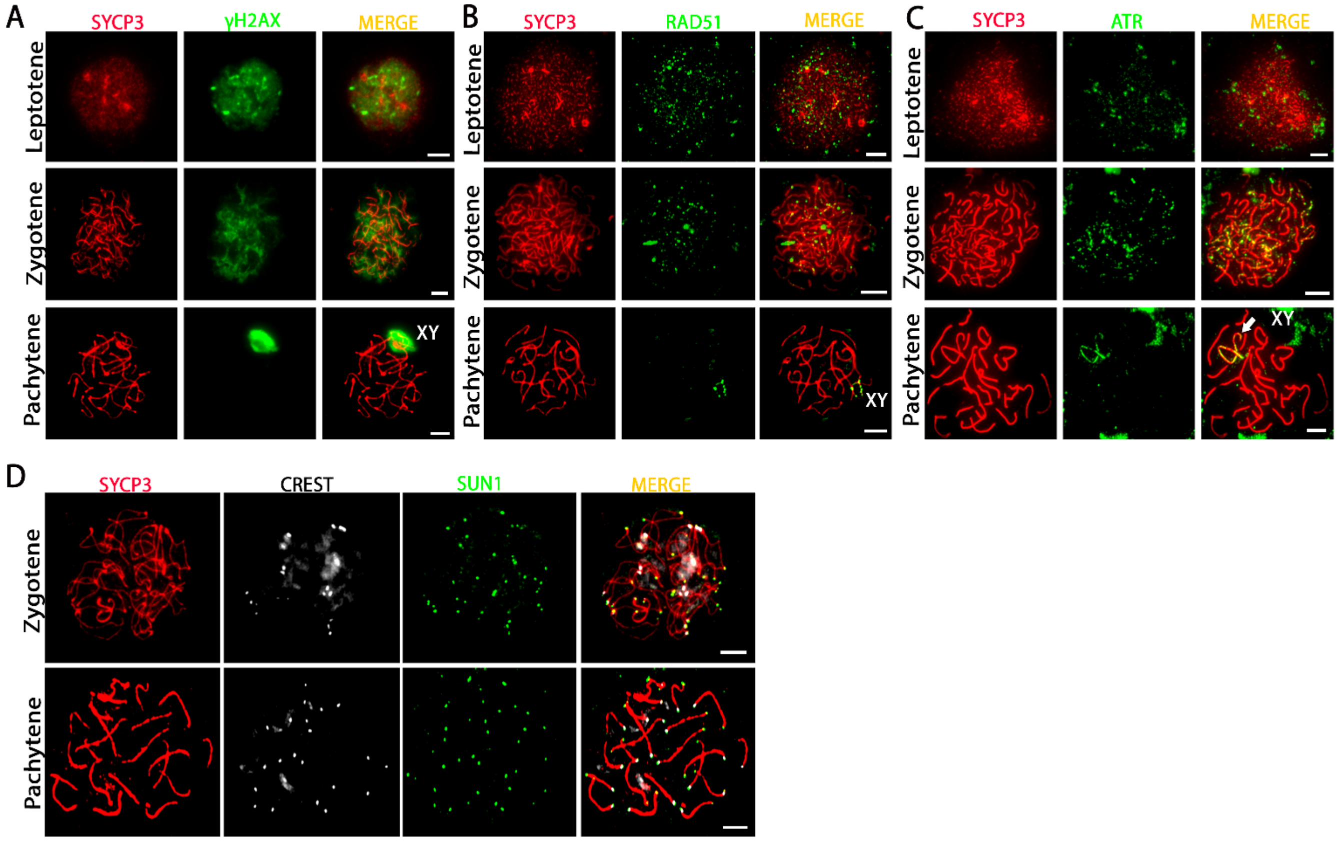

Figure S1. Positive control experiments using mouse testicular cells.

In vivo meiotic cells stained for SYCP3 (red) and **(A)** γH2AX (green), **(B)** RAD51 (green), **(C)** ATR (green), **(D)** SUN1 (green) and centromeres (CREST, white). Scale bar, 5µm.

Supplemental Experimental Procedures

Primary antibodies				
Target Protein	Host	Source	Cat. Number	IHC Dilution
γ-H2AX	Mouse	Merck Millipore	05–636	1:10,000
SYCP3	Mouse	Abcam	ab97672	1:600
SYCP3	Rabbit	NOVUS	NB300-231	1:2500
CREST-serum	Human	FitzGerald	90C-CS1058	1:600
ATR	Rabbit	Cell Signaling Technology	#2790	1:100
α-Tubulin	Mouse	Sigma	T9026	1:200
RAD51	Rabbit	Thermo Fisher Scientific	PA5-27195	1:100
MLH1	Mouse	BD Pharmingen	550838	1:50
SUN1	Guinea pig	Provided by Manfred Alsheimer	N/A	1:600
**Secondary antibodies**				
Fluorescence	Host	Source	Cat. Number	IHC Dilution
Alexa Fluor 488	Donkey anti-Mouse	Thermo Fisher Scientific	A21202	1:1000
Alexa Fluor 488	Donkey anti-Rabbit	Thermo Fisher Scientific	A21206	1:1000
Alexa Fluor 488	Goat anti-Guinea pig	Thermo Fisher Scientific	A11073	1:1000
Alexa Fluor 555	Goat anti-mouse	Thermo Fisher Scientific	A21424	1:1000
Alexa Fluor 555	Donkey anti-Rabbit	Thermo Fisher Scientific	A31572	1:1000
Alexa Fluor 647	Goat anti-Human	Thermo Fisher Scientific	A21445	1:1000

Table S1. Antibodies used in this study. Related to Figures 1, 3, 4, 5 and S1.

Supplemental Experimental Procedures

GS cells and Sertoli cell line culture

Mouse GS cells were cultured as previously reported (Kanatsu-Shinohara et al., 2003; Mulder et al., 2017; Zheng et al., 2017). After the third passage, the cells were cultured on mitotically inactivated mouse embryonic fibroblasts (MEFs; Gibco, A34962), using in a medium mainly composed of StemPro-34 SFM medium (Thermo Fisher Scientific), StemPro-34 Supplement (Thermo Fisher Scientific), 1% fetal bovine serum (FBS), recombinant human GDNF (10 ng/ml, 450-10, Peprotech), recombinant human bFGF (10 ng/ml, 100-18B, Peprotech), recombinant human EGF (20 ng/ml, AF-100-15, Peprotech), recombinant human LIF (10 ng/ml, CYT-644, Prospec), as well as other components as previously reported (Kanatsu-Shinohara et al., 2003). GS cells were refreshed every 2-3 days, dissociated by accutase (Thermo Fisher Scientific) and passaged every 5-7 days (doubling time, 3 days) at a ratio of 1:4-6 on fresh mitotically inactivated mouse embryonic fibroblasts. The cells were maintained at 37°C in 5% CO_2 in air. The cells used for this research were mostly at passage 20, with a maximum of 26 passages.

As a feeder cell, we used available Sertoli cell lines SK49 or TM4. SK49 was established by Walther et al., from 10-day-old male H-2Kb- tsA58 transgenic mice carrying an inducible temperature-sensitive SV40 T antigen (Walther et al., 1996). This cell line is able to express Sertoli cell-specific pattern markers and exhibit distinct Sertoli cell properties. TM4 was establishes by Matfier et al., from 11-13 days of age male BALB/c - nu/+ mice (Matfier, 1980). Both Sertoli cell lines were cultured at 37°C and 5% CO_2 in Dulbecco's Eagle's medium (DMEM; Thermo Fisher Scientific, Waltham, MA, USA) supplemented with 10% fetal bovine serum (FBS), penicillin (100 U/mL) and streptomycin (100 U/mL).

In vitro meiosis of GS cells

SK49 cells or TM4 Sertoli cells, inactivated by mitomycin (10µg/ml, M7949, Sigma), were grown on 12-well plates pre-coated with laminin (20 µg/ml, L2020, Sigma) to a density of 1 x10^5. Then GS cells were seeded on these Sertoli cells for two days to maintain GS cell proliferation as described previously (Kanatsu-Shinohara et al., 2003). To induce meiosis, day 0 to day 3 (Fig. 1A), the cells were cultured in a medium composed of StemPro-34 SFM medium and StemPro-34 Supplement, 10% KnockOut Serum Replacement (KSR), Retinoic acid (2µM, R2625, sigma), Recombinant Mouse BMP-4 Protein (20 ng/ml, 5020-BP, R&D Systems), Recombinant Mouse Activin A Protein (100 ng/ml, 338-AC, R&D Systems). From day 3 to day 9 after meiosis induction, the medium was composed of StemPro-34 SFM medium and StemPro-34 Supplement, 10% KSR, Bovine Pituitary Extract (BPE) (50µg/ml, 354123, Corning Life Sciences), Follicle-stimulating hormone (FSH) (200 ng/ml, F4021, Sigma), Testosterone (10 µM, 86500, Sigma) and refreshed daily.

Okadaic acid (OA)-induced generation of metaphase-like cells in vitro

For generation of metaphase-like cells *in vitro*, cells were cultured in Dulbecco's modified Eagle's medium (DMEM) supplemented with 5% FBS, 3 µM okadaic acid (OA; 459618, Millipore) at 34°C for 4 hours and fixed in 4% paraformaldehyde (PFA). For negative controls no OA was added.

Cytospins

The cells were detached from the culture dish using 0.25% trypsin and washed with 1x phosphate buffered saline (PBS) and diluted in 200 µL PBS/1% BSA containing 30,000 to 50,000 cells for each cytospin spot and spun for 7 minutes at 112g. The slides were air dried for 10min, fixed in 4% PFA and stored at 4°C in PBS or stored at -80°C after air drying.

Karyotyping

The Cells were dissociated into single cell suspension after 8 days after spermatogonial differentiation, subjected to hypotonic treatment with 75 mM KCl at 37 ◦C for 10 minutes and fixed in freshly prepared methanol/acetic acid (ratio: 3:1). Cells were dropped onto glass slides from 1 m distance and air dried. Chromosomes were stained with Hoechst 33342.

Flow Cytometry

For FACS analysis, after the cells were fixed and permeabilized in 70% Ethanol (EtOH). The cells were labeled with 1 µg/1 × 10^5 cells propidium iodide (PI, Sigma, P4964) in FACS/EDTA buffer (1× PBS with 1% FCS/0.1% NaN3/2 mM EDTA) containing DNAse-free RNAse A (v/v 1:20) for 5 min. The measurements and sorting were performed on a Sony SH800Z cell Sorter (Sony Biotechnology Inc. Japan). Data was analyzed using FlowJo software version 10. For acrosome detection, the sorted "haploid" peak (putative 1C-region) cells were incubated with lectin peanut agglutinin (PNA) conjugated with Alexa Fluor 488 conjugate (1:700, L21409, Life Technologies) for 15min.

Supplemental References:

Kanatsu-Shinohara, M., Ogonuki, N., Inoue, K., Miki, H., Ogura, A., Toyokuni, S., and Shinohara, T. (2003). Long-term proliferation in culture and germline transmission of mouse male germline stem cells. Biology of reproduction *69*, 612-616.

Matfier, J.P. (1980). Establishment and characterization of two distinct mouse testicular epithelial cell lines. Biology of reproduction *23*, 243-252.

Mulder, C.L., Catsburg, L.A.E., Zheng, Y., de Winter-Korver, C.M., van Daalen, S.K.M., van Wely, M., Pals, S., Repping, S., and van Pelt, A.M.M. (2017). Long-term health in recipients of transplanted in vitro propagated spermatogonial stem cells. Human reproduction *33*, 81-90.

Walther, N., Jansen, M., Ergün, S., Kascheike, B., and Ivell, R. (1996). Sertoli Cell Lines Established fromH-2Kb-tsA58 Transgenic Mice Differentially Regulate the Expression of Cell-Specific Genes. Experimental cell research *225*, 411-421.

Zheng, Y., Jongejan, A., Mulder, C.L., Mastenbroek, S., Repping, S., Wang, Y., Li, J., and Hamer, G. (2017). Trivial role for NSMCE2 during in vitro proliferation and differentiation of male germline stem cells. Reproduction *154*, 81-95.
